# Islet Biology and Metabolism

**DOI:** 10.3390/metabo11110786

**Published:** 2021-11-18

**Authors:** Belinda Yau, Melkam A. Kebede

**Affiliations:** School of Medical Sciences, Faculty of Medicine and Health, Charles Perkins Centre, University of Sydney, Camperdown, Sydney 2006, Australia; belinda.yau@sydney.edu.au

This Special Issue, *Islet Biology and Metabolism*, was intended as a collection of studies highlighting the importance of the pancreatic islet—in both form and function—to our growing understanding of metabolic physiology and disease.

The pancreatic islets of Langerhans are composed of five distinct secretory cell types that influence metabolism via the secretion of carefully balanced mixtures of islet hormones into the circulation. By mass, the beta-cells contribute to 60–70% of the islet and are responsible for the secretion of the only glucose-lowering hormone insulin [1]. Alpha-cells, which secrete glucagon, make up another 20–30% of the islet, with delta-cells, epsilon-cells, and PP-cells, which secrete somatostatin, ghrelin, and pancreatic polypeptide, respectively, representing the approximate 10% remaining [1] endocrine cell types. As beta-cells are the primary cell type of the islet, islet function is often studied correlatively with beta-cell function, which in turn is often studied synonymously with insulin secretion.

In most species, including humans, insulin secretion is bi-phasic, with a rapid first phase followed by a slower but sustained second phase of secretion. These distinct phases of secretion are mediated by two pathways. The first, triggered by nutrient or glucose-stimulated closure of the ATP-sensitive K^+^-channels and subsequent depolarisation-induced influx of calcium, is termed the “triggering pathway” [2]. The second, termed the “amplifying pathway”, which relies on metabolic amplification of the initial stimulus originating at the mitochondria, is reviewed in this Special Issue by Rustenbeck et al. [3]. Gerber et al. also presently reported on dose-dependent responses within mouse and human islets with extremely high glucose concentrations driven by the amplifying pathway—outcomes that have been unappreciated despite comparable clinical observations [4]. Relatedly, the role of mitochondrial metabolism is further highlighted in a study by Kabra et al., who utilised a diet-induced obesity model to correlate and classify properties of islet mitochondrial respiration with respect to glucose-stimulated insulin secretion in the islet [5].

Zooming in deeper within the beta-cell, we approach the subcellular compartments inside which insulin is synthesised, processed, and stored. These vesicles, termed insulin secretory granules, and the proteins involved in their formation, maturation, and secretion, are comprehensively discussed by Germanos et al. [6] within this Special Issue. This article is further complemented by a review on the technical advances and limitations in the isolation of insulin secretory granules for analysis by Norri et al. [7], which reflects particularly on knowledge gaps in the field with respect to insulin granule proteomics.

As we continue to advance our characterisation of the beta-cell and the islet, new techniques and technologies are becoming available. The use of machine learning to augment our analyses is showcased in a study by Cottle et al. [8], which demonstrated the utility of deep learning to model 3D pancreatic islets and measure subcellular proteins of beta-cells within pancreatic slices. As these analyses show their capability to identify cellular polarity within islets—a phenomenon closely associated to beta-cell regulated secretory behaviour [9]—they further highlight their potential to make functional assessments of whole islets in situ.

Indeed, a better understanding of the functional islet in its native environment is especially critical to our understanding of the progression of disease. For example, it is well known that maternal nutrition plays an important role in programming beta-cell development and the function of her offspring. In a detailed review, O’Hara et al. discussed our current understanding of fetal exposure to either maternal caloric excess or nutrient restriction. Significantly, they discussed the effects of fetal malnutrition with respect to multiple outcomes, from mitochondrial metabolism to islet morphology and beta-cell function and the consequences for Type 2 Diabetes development [10]. 

In Type 1 Diabetes (T1D), autoimmune-destruction of the pancreatic beta-cells results in insulin insufficiency [11], and islet transplantation is an established approach to beta-cell replacement therapy for patients with T1D. This involved isolating islets from the pancreas of a deceased donor and implanting them into a T1D patient. Although recent advances in islet isolation and culture techniques have improved the quality of transplant islets and thus the outcomes of patients, there are still several experimental and logistical issues that could be optimised. Presently, Hawthorne et al. discussed the implications and outcomes for islet transplant across large distances with respect to the national islet transplant network in Australia [12]. Furthermore, in an experimental mouse transplant model, Leibiger et al. demonstrated a proof-of-concept technique that allows the expression and functional action of a non-native hormone in intraocular transplanted pseudo-islets [13].

T1D pathology is further reviewed in this Special Issue in the context of the gut microbiome by Priyadarshini et al., who particularly implicated short-chain fatty acid receptors as potential targets for therapy [14]. Additionally, two research articles focused on the prevention of T1D incidence and progression using the spontaneous diabetic NOD mouse model. The first, by Waters et al., investigated the role of the SLC6A19 amino acid transporter in the development of T1D using SLC6A19-deficient female NOD mice [15], and the second, by Borg et al., assessed the benefit of the anti-advanced glycation end products drug Alagebrium Chloride as a pre-diabetic therapy and its subsequent effects on pancreatic function [16].

Altogether, these articles present a high-quality perspective of both innovative and established islet biology research. As guest editors, we would like to thank all the authors for their noteworthy studies, the peer reviewers for their assessments and comments for the refinement of these articles, and the Metabolites Editorial Office for their support and contributions to this *Islet Biology and Metabolism* Special Issue.
